# Hypertonic sodium lactate infusion reduces vasopressor requirements and biomarkers of brain and cardiac injury after experimental cardiac arrest

**DOI:** 10.1186/s13054-023-04454-1

**Published:** 2023-04-22

**Authors:** Filippo Annoni, Fuhong Su, Lorenzo Peluso, Ilaria Lisi, Enrico Caruso, Francesca Pischiutta, Elisa Gouvea Bogossian, Bruno Garcia, Hassane Njimi, Jean-Louis Vincent, Nicolas Gaspard, Lorenzo Ferlini, Jacques Creteur, Elisa R. Zanier, Fabio Silvio Taccone

**Affiliations:** 1 Department of Intensive Care, Erasme Hospital, Lennik Road 808, 1070 Brussels, Belgium; 2 Experimental Laboratory of Intensive Care, Free University of Brussels, Brussels, Belgium; 3 Department of Biomedical Sciences, Humanitas University, Pieve Emanuele, Milan, Italy; 4 Department of Anesthesiology and Intensive Care, Humanitas Gavazzeni, Via M Gavazzeni 21, 24125 Bergamo, Italy; 5 Laboratory of Traumatic Brain Injury and Neuroprotection, Department of Acute Brain Injury, Istituto di Ricerche Farmacologiche Mario Negri IRCCS, Via Mario Negri 2, 20156 Milan, Italy; 6 Department of Neurology, Erasme Hospital, Lennik Road 808, 1070 Brussels, Belgium; 7 Neurology Department, School of Medicine, Yale University, New Haven, CT USA

**Keywords:** Cardiac arrest, Resuscitation, Post-arrest, Anoxic injury, ischemia–reperfusion

## Abstract

**Introduction:**

Prognosis after resuscitation from cardiac arrest (CA) remains poor, with high morbidity and mortality as a result of extensive cardiac and brain injury and lack of effective treatments. Hypertonic sodium lactate (HSL) may be beneficial after CA by buffering severe metabolic acidosis, increasing brain perfusion and cardiac performance, reducing cerebral swelling, and serving as an alternative energetic cellular substrate. The aim of this study was to test the effects of HSL infusion on brain and cardiac injury in an experimental model of CA.

**Methods:**

After a 10-min electrically induced CA followed by 5 min of cardiopulmonary resuscitation maneuvers, adult swine (*n* = 35) were randomly assigned to receive either balanced crystalloid (controls, *n* = 11) or HSL infusion started during cardiopulmonary resuscitation (CPR, Intra-arrest, *n* = 12) or after return of spontaneous circulation (Post-ROSC, *n* = 11) for the subsequent 12 h. In all animals, extensive multimodal neurological and cardiovascular monitoring was implemented. All animals were treated with targeted temperature management at 34 °C.

**Results:**

Thirty-four of the 35 (97.1%) animals achieved ROSC; one animal in the Intra-arrest group died before completing the observation period. Arterial pH, lactate and sodium concentrations, and plasma osmolarity were higher in HSL-treated animals than in controls (*p* < 0.001), whereas potassium concentrations were lower (*p* = 0.004). Intra-arrest and Post-ROSC HSL infusion improved hemodynamic status compared to controls, as shown by reduced vasopressor requirements to maintain a mean arterial pressure target > 65 mmHg (*p* = 0.005 for interaction; *p* = 0.01 for groups). Moreover, plasma troponin I and glial fibrillary acid protein (GFAP) concentrations were lower in HSL-treated groups at several time-points than in controls.

**Conclusions:**

In this experimental CA model, HSL infusion was associated with reduced vasopressor requirements and decreased plasma concentrations of measured biomarkers of cardiac and cerebral injury.

**Supplementary Information:**

The online version contains supplementary material available at 10.1186/s13054-023-04454-1.

## Introduction

Sudden cardiac arrest (CA) remains a major healthcare burden worldwide, being a leading cause of morbidity and mortality [1]. Even when cardiopulmonary resuscitation (CPR) succeeds and return of spontaneous circulation (ROSC) is achieved, survivors remain at high risk of death in the days following the arrest and only a minority return to a quality of life similar to that prior to the event [2]. Among patients admitted to the hospital after resuscitation from CA, extensive cardiac and brain injuries represent the leading cause of unfavorable outcomes, with brain injury accounting for the majority of deaths in these patients [3]. Various drugs and interventions, including targeted temperature management, have been tried in these patients but none has been proved to improve neurological outcomes [4–7].

In patients with traumatic brain injury, hypertonic sodium lactate (HSL) has been shown to be safe, to reduce intracranial pressure (ICP), to prevent episodes of raised ICP, to improve cerebral metabolism, and to have a cerebral glucose sparing effect [8–13]. Moreover, HSL infusion may improve cardiac performance in some selected populations, such as patients with acute heart failure [14, 15]. As such, HSL infusion could represent a potential therapeutic option in CA patients. In particular, HSL could: (1) provide an alternative energy substrate for neurons in a situation of metabolic stress; (2) reduce glutamate-related excitotoxicity; (3) mitigate severe post-resuscitation metabolic acidemia; (4) decrease ICP and cellular swelling; and (5) improve post-resuscitation myocardial dysfunction [16].

HSL administration has only been studied in one rabbit model of CA, in which it increased mean arterial pressure, improved cardiac function, improved cerebral mitochondrial function, and reduced brain injury as assessed by reduced plasma levels of the protein S100ß [17]. Large animal models are better suited for research in CA, enabling use of mechanical CPR, frequent blood sampling, and invasive monitoring devices. Furthermore, pigs have a gyrencephalic brain architecture and a cardiovascular system that closely resemble those in humans, increasing the translatability of experimental findings.

We postulated that HSL infusion could decrease cerebral and cardiac injury after CA. We therefore assessed the effects of HSL infusion during and after CPR on the cardiovascular system, on brain function and perfusion, and on related biomarkers of organ injury.

## Methods

### Experimental procedure

The Institutional Review Board for Animal Care of the Free University of Brussels (Belgium) approved all experimental procedures (number of Ethical Committee approval: 704 N), which were also in compliance with the ARRIVE (Animal Research: Reporting in Vivo Experiments, Additional file 1: Table S1) guidelines. Care and handling of the animals were in accord with National Institutes of Health guidelines (Institute of Laboratory Animal Resources).

A detailed description of this experimental model has been published previously [18], and full information regarding animal handling is provided in Supplementary Material. Briefly, on the day of the experiment the animal (adult swine, both sexes) was initially sedated in the cage and then transported to the operating room. An arterial catheter was inserted in the femoral artery and a peripheral venous catheter sited. Endotracheal intubation was then performed, and mechanical ventilation started. The animal was sedated with a 1% mixture of inhaled sevoflurane. It was then equipped with a Foley catheter, a three-lumen central venous line in the external jugular vein, a pulmonary artery catheter, and a single lumen central venous catheter inserted upstream to allow sampling of brain effluent blood. After pronation, multimodal neuromonitoring devices were placed surgically, including two cerebral microdialysis (CMD) catheters (one in each frontal lobe), a single probe measuring ICP, cerebral temperature, and brain tissue oxygen tension (PbtO_2_) in one parietal lobe, a laser Doppler probe in the other parietal lobe, and one stereoelectroencephalography (sEEG) wire in each parietal lobe. The animal was turned supine, and after stabilization, ventricular fibrillation was induced electrically via a pacing wire and left untreated for 10 min. Chest compressions were then started at a rate of 100/min for 5 min and ventilation resumed with a FiO_2_ of 1, along with sedation and analgesia. After one minute, an intravenous injection of epinephrine was administered. At the end of the 5-min period, a biphasic electric countershock was delivered. CPR was restarted for an additional minute in case of unsuccessful biphasic shock, and another shock was delivered every minute along with a second dose of epinephrine for a total CPR time of seven min. ROSC was considered to have been achieved if the cardiac rhythm was associated with a mean arterial pressure (MAP) > 65 mmHg for at least 20 min. If the animal failed to achieve ROSC after 12 shocks, it was considered dead. If ROSC was achieved, the animal was placed in the prone position again and observed for an additional 12 h. Death was confirmed by ventricular fibrillation waves on the electrocardiogram (ECG) and a concomitant decrease in arterial blood pressure. All animals received targeted temperature management aimed at a temperature of 34 °C.

### Group allocation and treatment preparation

**Fig. 1:**
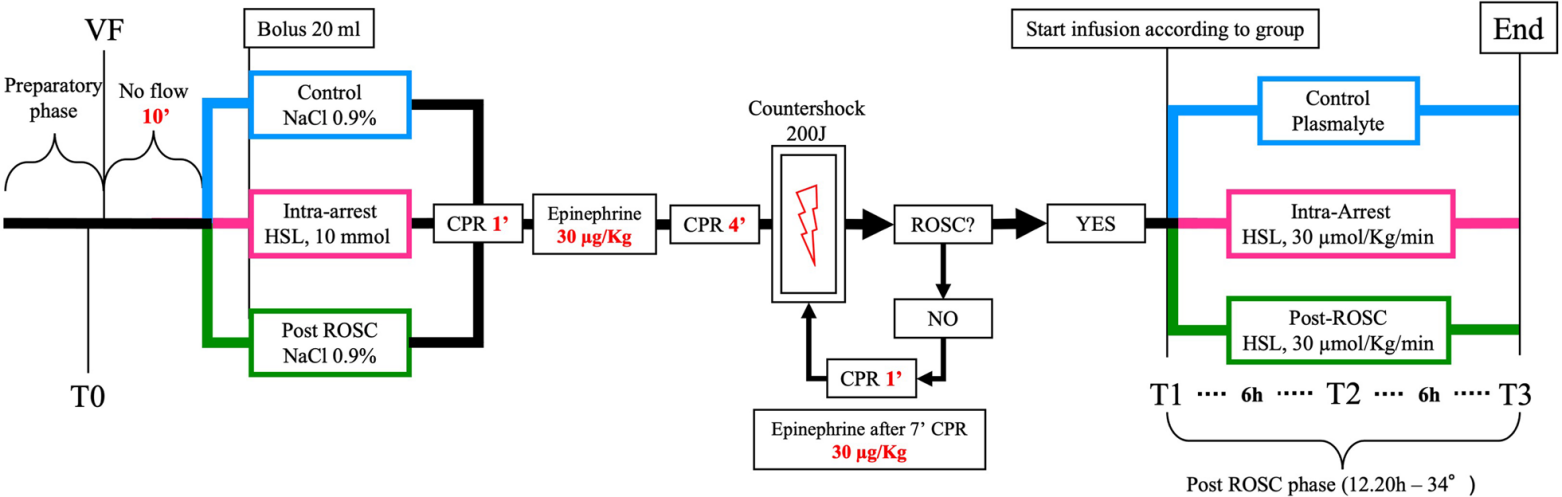
Timeline of the experiment. Control group is shown in blue, Intra-arrest group in pink, and Post-ROSC group in green. VF = Ventricular fibrillation; CPR = cardiopulmonary resuscitation; ROSC = return of spontaneous circulation; and T0-3 = blood sampling time points

On the day of the experiment, animals were randomly assigned (simple randomization) to receive a bolus of 20 mL of NaCl 0.9% at the beginning of CPR maneuvers and a continuous infusion of balanced crystalloids during the observation period (control group), an intravenous bolus of 10 mmoL of HSL (Monico SpA, Venezia, Italy) diluted in 20 mL of NaCl 0.9% at CPR initiation followed by a continuous infusion of 30 µmol/Kg/min during the observation period (Intra-arrest group), or 20 mL of NaCl 0.9% at the beginning of CPR followed by a continuous infusion of 30 µmol/Kg/min HSL during the observation period (Post-ROSC group, Fig. 1). Due to the magnitude of the changes in pH and lactate, blinding was not feasible. In the HSL-treated groups, specific safety limits were pre-established to standardize the administration of HSL and reproduce conditions similar to clinical practice. Specifically, each hour, the HSL perfusion rate was reduced by 20% if any of the following criteria were met: arterial pH increases > 0.10 compared to baseline pH or > 7.65; Na^+^ ≥ 155 mEq/l; or plasma Osm ≥ 320 mOsm.

Throughout the experiment, a minimal MAP of at least 65 mmHg was maintained using titrated norepinephrine doses. All animals received a fixed continuous infusion of balanced crystalloids (5–10 ml/kg/h); additional fluids were administered to keep the pulse pressure variation (PPV) < 14%.

Hyperglycemia after ROSC was left uncorrected if the blood glucose concentration was < 300 mg/dl within the first hour after ROSC and < 250 mg/dl during the study period, and otherwise corrected with an infusion of 10U of regular insulin (Actrapid, Novo Nordisk, Bagsværd, Denmark).

### Blood and brain interstitial fluid sampling

Arterial blood samples were obtained prior to CA induction (T0), 20 min after ROSC (sustained ROSC, T1), and 6 (T2) and 12 h later (T3). They were immediately centrifuged and separated for plasma to be stored (10 mL) at − 80 °C. Arterial and jugular blood gas analyses were carried out before and after endotracheal intubation, at T0 and T1, and then hourly until the end of the experiment; the arterio-jugular differences for lactate and glucose were computed at each time point. Mixed central venous blood was collected at T0, T1, and then every three hours to allow instrument calibration. CMD sampling was performed at T0 and every hour after T1 from the same catheter. The spare CMD sample from the second CMD catheter was stored at − 80 °C in case of necessity.

### Brain tissue collection

After animal sacrifice (using an intravenous injection of 80 mEq of potassium chloride under deep anesthesia), the frontal and parietal bones were carefully opened with the help of a Kerrison bone punch. The dura mater was dissected with a scalpel, and one section of the parietal cortex of approximately 0.5 cm^3^ was harvested on each side and immediately frozen in liquid nitrogen and stored at − 80 °C.

### Gene expression analysis

Total RNA was extracted from the cortex with a Ribospin II kit (GeneAll, #314-103). Samples were reverse transcribed with random hexamer primers using Multi-Scribe Reverse Transcriptase (Life Technologies). Real-time reverse transcription PCR was done with *RPL27* as the housekeeping gene. Relative gene expression was determined by the ΔΔCt method [19]. Data are expressed as the log2-fold difference compared to the control group. Genes and primer sequences are listed in Additional file 1: Table S2. The exploratory analysis included the following genes: microtubule-associated protein 2 (*MAP2*); glial fibrillary acid protein (*GFAP*); cluster of differentiation molecule 11ß (*CD11ß*); platelet and endothelial cell adhesion molecule 1 (*PECAM-1*); caspase 3 and 8 (*CASP-3* and *CASP-8*), and heme oxygenase 1 (*HO-1*). These genes were chosen to provide a representative picture of the tissue reaction to the injury from multiple viewpoints, including structural cellular integrity, inflammation, apoptosis, endothelial function, and oxidative stress.

### Brain injury biomarkers

Plasma concentrations of GFAP, neurofilament light chain protein (NFL), and neuron-specific enolase (NSE) were measured using commercially available single molecule array assay kits on an SR-X Analyzer [GFAP (#102336), NFL (#103400), and NSE (#102475)] as described by the manufacturer (Quanterix, Billerica, MA). A single batch of reagents was used for each analyte.

### Continuous intracerebral EEG monitoring

All sEEG traces acquired via the dedicated device and transferred to the acquisition software considered as baseline a 10-min window prior to the beginning of the CA induction (T0). During the CA procedure (i.e., prone position, no-flow time, low-flow time, and countershock), the sEEG electrodes were disconnected to prevent electrical damage of the experimental tools from the shock and reconnected as soon as possible after ROSC. The recording lasted throughout the observation phase (i.e., 12 h). The EEG signal with the best signal-to-noise ratio was selected for further analysis. All analyses were performed offline, using built-in and custom functions in MATLAB. We filtered the EEG signal between 1 and 15 Hz (4th-order Butterworth bandpass filter, filtfilt function in MATLAB) and then computed the Hilbert transform (Hilbert function in MATLAB) of the filtered signal and extracted the amplitude. We calculated the mean, kurtosis, skewness, and standard deviation of the amplitude using a sliding 1 min window with 50% overlap. The EEG background at T3 [20] was assessed for the presence of a suppressed background or burst suppression by two independent neurophysiologists, expert in EEG reading and blinded to the study group assignment.

### Data handling and reduction

Multiple variables were recorded continuously with a sampling frequency between 1 and 100 Hz, including blood pressure (systolic, diastolic, mean), heart rate, ICP, PbtO_2_, brain temperature, and cerebral blood flow (CBF). Data were extracted as means over periods of 60 s and successively reduced to means over 10 min. For the sEEG signal, the amplitude of the Hilbert transform of the filtered signal between 1 and 15 Hz was computed. Outliers were detected and eliminated using the ROUT method with a *Q* = 1% (where Q is the maximum desired false discovery rate).

### Statistical analysis

With an expected survival rate of > 90% [19] and given the absence of prior studies on the subject, an a priori convenient sample size of 10 animals per group was considered adequate for the study purposes. Continuous variables are expressed as means with standard deviation or medians with interquartile range, according to the population distribution, and discrete variables are expressed as percentages with 95% confidence intervals. Categorical variables were compared using Fisher’s exact test or a chi-square test, as appropriate. For multiple group comparison, ANOVA test, Welch’s test, Kruskal–Wallis test, or Dunnett’s test was used, as appropriate. To compare the time-based variations among groups, a linear mixed-effect model fitted for restricted maximum likelihood estimation (REML) was used. Two analyses were conducted for cerebral lactate and lactate-to-pyruvate ratio (LPR) (between T0 and T2 and between T2 and T3), and for the EEG variables (between T1 and T2 and between T2 and T3). EEG data from the treated groups were analyzed as aggregate over the observation period. For variation in gene expression compared to the control group, a one sample t test or Wilcoxon test was used, as appropriate. A value of *p* < 0.05 was considered statistically significant. Data analyses were performed using GraphPad Prism (version 9.3.1 for Macintosh, GraphPad Software, La Jolla, CA, USA) and MATLAB (version R2019b, The MathWorks Inc., Natick, MA, USA).

## Results

Of the 35 animals that underwent CA induction, 34 (97.1%) achieved ROSC (one animal in the Control group did not) and were included in the analysis: 11 in the control group, 12 in the Intra-arrest group, and 11 in the Post-ROSC group. One animal in the Intra-arrest group had a second VF episode 7 h after ROSC, possibly related to mobilization of the pulmonary artery catheter: despite a successful countershock, data acquired beyond this time point, as well as the brain tissue samples, were excluded from the analysis.

**Fig. 2:**
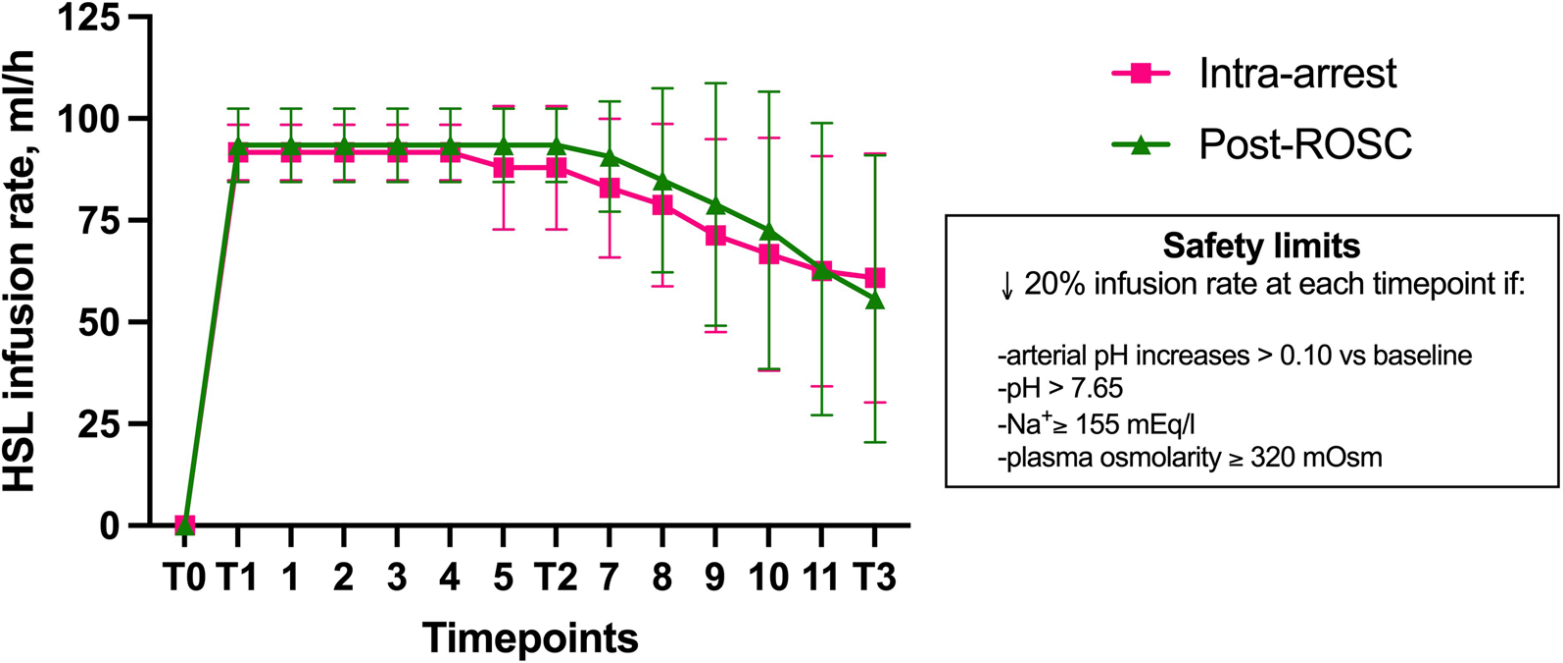
HSL infusion rate in treated groups. (Means ± SD)

Baseline characteristics of the study groups are shown in Table 1. There were no significant differences among the three groups in weight and sex, or time to target core temperature of 34 °C (3.9 ± 0.6 h; *p* = 0.89). In the Intra-arrest and Post-ROSC groups, there was a similar decrease in HSL infusion rate from the fifth hour Post-ROSC, according to the pre-specified safety protocol (Fig. 2).

**Table 1:** Baseline characteristics of the study groups

Variable	Group			*p* value
	Control (*n* = 11)	Intra-arrest (*n* = 12)	Post-ROSC (*n* = 11)	
Male, *n* (%)	8 (72)	6 (50)	6 (54)	0.51
Weight, Kg	47.9 (5.8)	50.3 (6.2)	51 (7.1)	0.47
Temperature, °C	38.0 (0.8)	37.8 (0.7)	37.4 (0.8)	0.19
Arterial pH	7.51 (0.03)	7.49 (0.02)	7.49 (0.02)	0.36
Arterial lactate, mmol/L	1.3 (0.3)	1.1 (0.2)	1.5 (0.4)	0.06
Arterial glucose, g/dl	112.5 (31.7)	97.0 (16.3)	106.8 (13.1)	0.24
P/F	435.7 (57.3)	454.3 (68.0)	482.4 (69.7)	0.25
PaCO_2_, mmHg	41.6 (3.1)	43.0 (1.7)	41.2 (4.0)	0.34
Na^+^, mmol/L	133.4 (2.37)	134.6 (3.5)	135.9 (5.3)	0.36
K^+^, mmol/L	3.8 (0.3)	3.7 (0.1)	3.7 (0.3)	0.27
Cl^−^, mmol/L	100.7 (1.5)	101.8 (2.2)	103.2 (5.0)	0.21
Base excess, mEq/L	9.0 (3.0)	8.5 (1.1)	7.7 (2.3)	0.45
Osmolarity, osm/L	268.5 (3.2)	269.7 (6.9)	272.6 (10.2)	0.43
MAP, mmHg	83 (12)	89 (12)	87 (11)	0.57
HR, bpm	87 (14)	105 (29)	100 (22)	0.22
CVP, mmHg	7.3 (3.2)	6.6 (3.2)	8.4 (3.3)	0.47
SvO_2_, %	60.8 (7.9)	63.3 (7.3)	68.2 (6.9)	0.15
CO, L/min	5.8 (1.1)	6.0 (1.3)	5.3 (0.5)	0.28
PPV, %	11.4 (1.8)	11.6 (3.5)	12.8 (2.7)	0.50
PCWP, mmHg	10.5 (2.9)	9.5 (3.1)	11.5 (3.1)	0.29
CPO, Watt	0.8 (0.3)	1.1 (0.3)	0.9 (0.1)	0.11
DO_2_, ml/min	624.5 (180.5)	688.7 (193.3)	608.7 (77.3)	0.48
VO_2_, ml/min	245.7 (93.5)	237.2 (65.7)	190.2 (74.6)	0.23
OER, %	39.4 (6.3)	36.7 (8.6)	31.0 (11.1)	0.09
*Diuresis, ml	600 (256–825)	600 (300–700)	600 (500–700)	0.95
*ICP, mmHg	8.0 (6.1–10.3)	12.6 (6.8–14.4)	10.1 (7.0–12.8)	0.31
*PbtO_2_, mmHg	33.2 (31.9–38.3)	32.5 (25.7–41.6)	40.1 (24.8–42)	0.40
Hemoglobin, g/dl	8.9 (0.7)	8.4 (0.7)	8.4 (0.6)	0.20

Data are reported as mean (SD) or *median (IQR)

Data are reported as mean (SD). P/F = ratio between partial arterial oxygen pressure and fraction of inspired oxygen; PaCO_2_ = arterial partial pressure of carbon dioxide; MAP = mean arterial pressure; HR = heart rate; CVP = central venous pressure; SvO_2_ = mixed venous oxygen saturation; CO = cardiac output; PPV = pulse pressure variation; PCWP = pulmonary capillary wedge pressure; CPO = cardiac power output; DO_2_ = oxygen delivery; VO_2_ = oxygen consumption; OER = oxygen extraction ratio; ICP = intracranial pressure; and PbtO_2_ = brain tissue oxygen pressure

### Cardiopulmonary resuscitation

CPR lasted 300 (300–360) seconds in all groups. There were no significant differences among groups in maximal end-tidal CO_2_ (etCO_2_) over the first minute, the required number of shocks, the epinephrine doses administered, or the incidence of arrhythmia during the first 30 min after ROSC (Additional file 1: Table S3).

### Physiological and metabolic variables

**Fig. 3:**
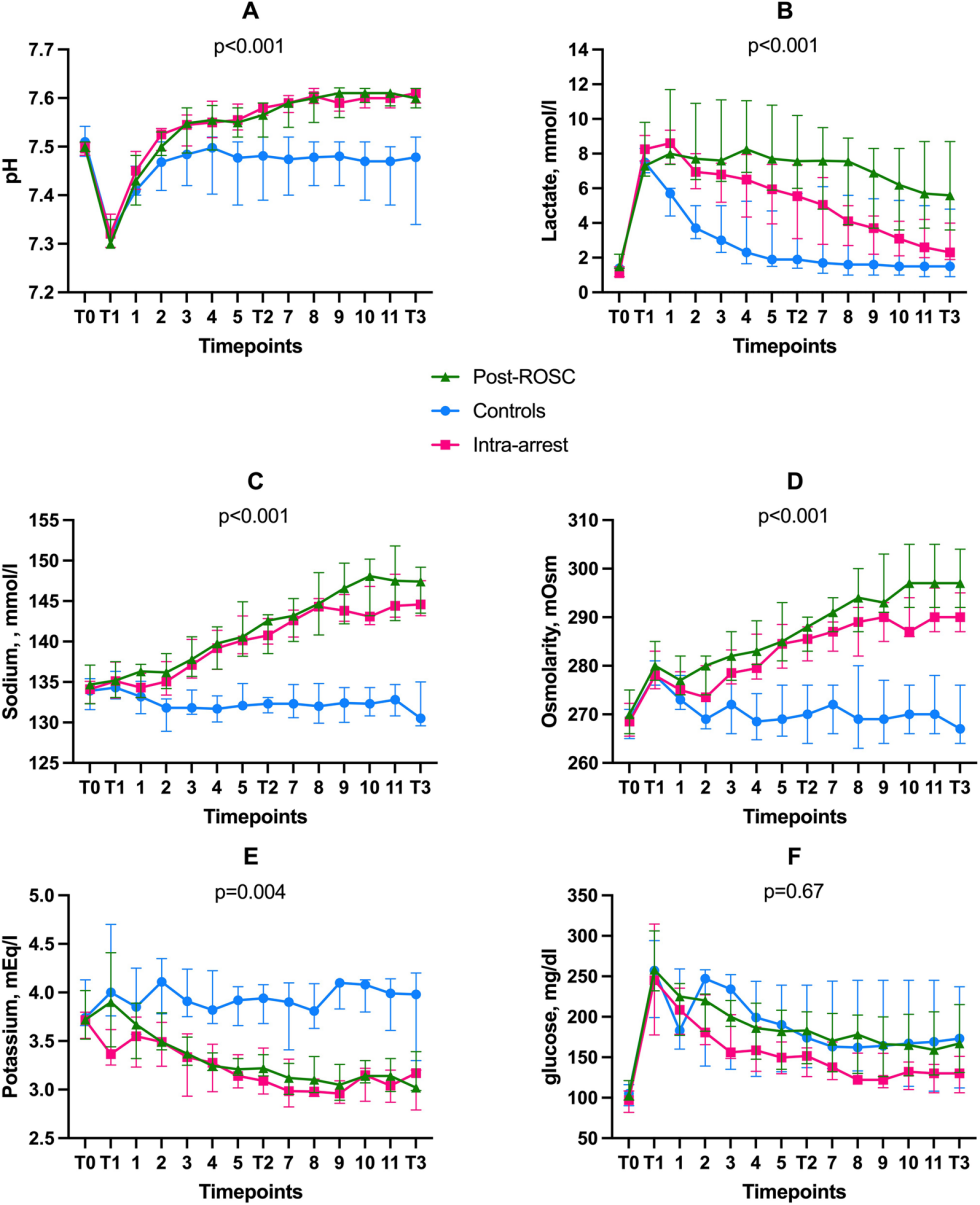
Time course of main physiological variables and arterial electrolyte concentrations. **A** Arterial pH; **B** arterial lactate; **C** sodium; **D** osmolarity; **E** potassium; and **F** arterial glucose. Data are shown as medians with interquartile ranges (25th–75th)

After ROSC, arterial pH was statistically significantly higher in the HSL groups than in the control group (*p* < 0.001), as were the arterial lactate (*p* < 0.001) and sodium (*p* < 0.001) concentrations, and the arterial plasma osmolarity (*p* < 0.001); potassium concentrations were lower (*p* = 0.004; Fig. 3). Arterial glucose concentrations did not differ among the groups; only one animal in the control group required 10 units of intravenous insulin for hyperglycemia, two hours after ROSC.

The arterio-jugular difference in glucose was lower and the arterio-jugular difference in lactate higher in both HSL groups compared to controls (*p* < 0.001 and *p* < 0.001, respectively; Additional file 1: Fig. S1). Despite a different trajectory, PaCO_2_ remained within the desired range and was similar in all groups (*p* = 0.02 for interaction, *p* = 0.07 for groups).

### Hemodynamic variables

**Fig. 4:**
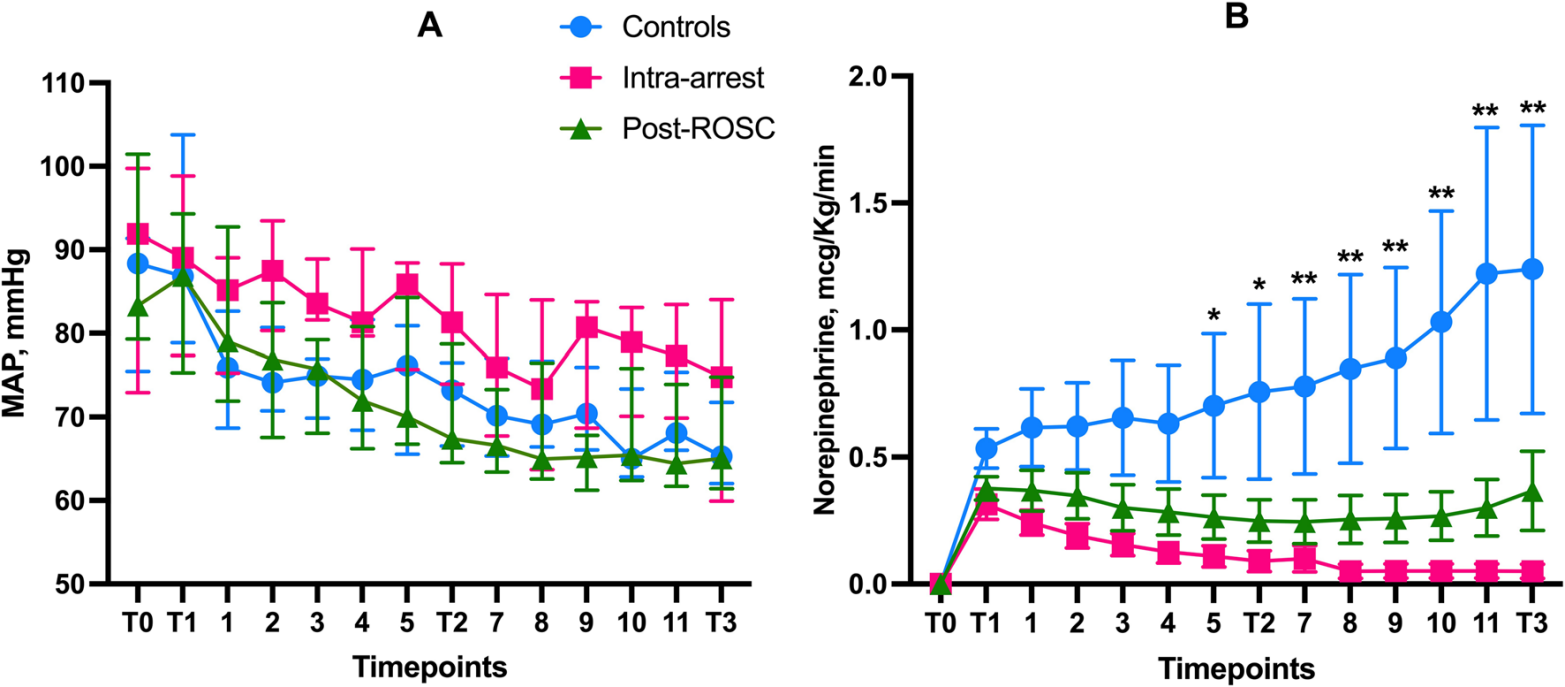
Mean arterial pressure (median with interquartile range, **A**) and norepinephrine requirements (means and SEM, **B**) in the three groups. *Significant difference Intra-arrest group versus controls; ** significant difference both HSL groups versus controls

Significantly lower norepinephrine doses were required to maintain the target MAP ≥ 65 mmHg in the HLS groups than in the controls (*p* = 0.005; Fig. 4); other hemodynamic variables, including cardiac output, central venous pressure, heart rate, PPV, and pulmonary artery wedge pressure, were similar across the groups (Additional file 1: Table S4). Despite a similar total fluid balance among groups (*p* = 0.36), urine output was greater in the HSL groups than in controls on the last 4 h of the observation period (*p* < 0.01, Additional file 1: Fig. S2).

### Multimodal neuromonitoring

ICP, cerebral perfusion pressure (CPP), and PbtO_2_ remained within normal ranges at most time points with no significant differences among groups; there was no increase over time in the regional CBF assessed by laser Doppler and no significant differences among groups (Additional file 1: Fig. S3).

Cerebral lactate levels were higher in the HSL groups than in the controls (*p* = 0.004) in the first hours after ROSC, whereas cerebral glucose, pyruvate, LPR, glutamate, and glycerol levels were similar among groups over time (Additional file 1: Fig. S4).

### Plasma biomarkers

**Fig. 5:**
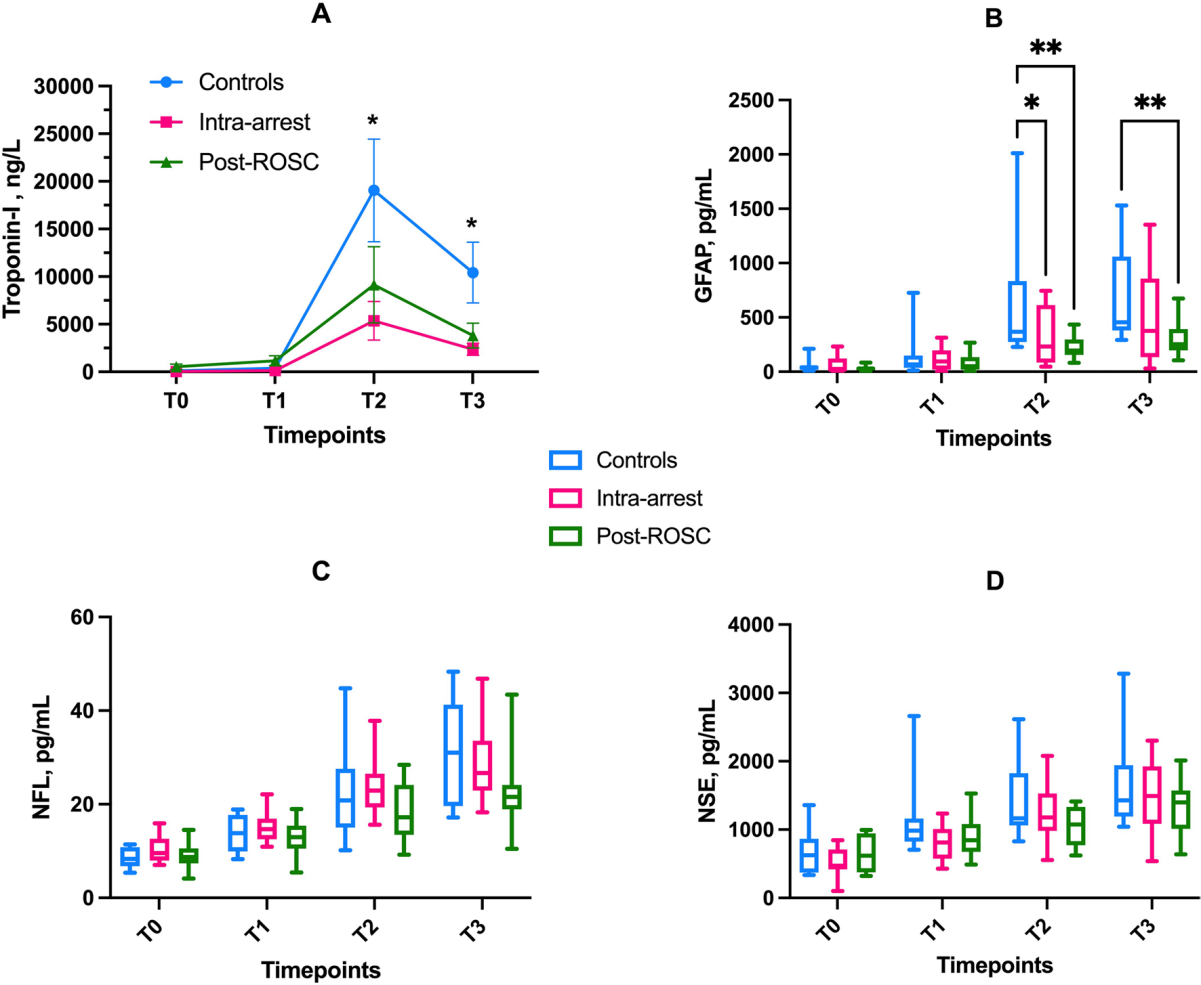
Plasma cerebral and cardiac biomarkers. **A** troponin I (means and SEM *: significantly different comparison Intra-arrest and Post-ROSC groups and Controls); **B** glial fibrillary acid protein; **C** neurofilament light chain; and **D** neuron-specific enolase. Boxes represent median values and interquartile ranges (25th–75th), whiskers extend between minimum and maximum values. *p < 0.05; **p < 0.01; ***p < 0.001

Circulating levels of troponin I were lower in the HSL groups than in the controls at T2 and T3 (Fig. 5A). There were no statistically significant differences in most of the other measured biomarkers, except for lactate dehydrogenase and alkaline phosphatase, which were lower in the HSL groups at T2 and T3 (Additional file 1: Fig. S5). For the brain injury biomarkers, circulating GFAP concentrations were lower in the HSL groups than in controls at T2 and T3. NFL and NSE values tended to be lower in the HSL groups, although this difference did not reach statistical significance (*p* = 0.14 and *p* = 0.39, respectively, Fig. 5C, D).

### sEEG

**Fig. 6:**
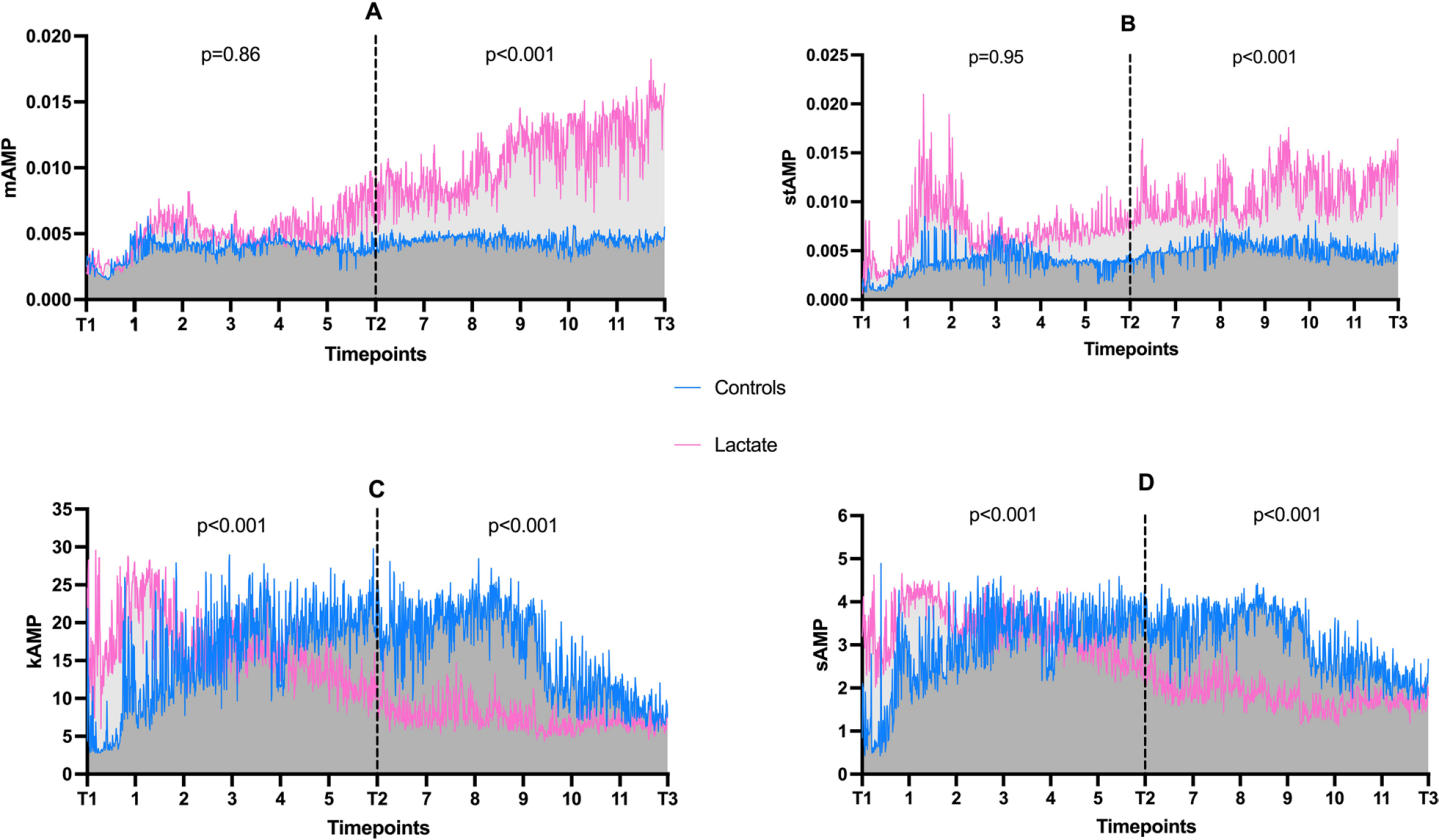
EEG over time; **A** mean amplitude; **B** mean standard deviation; **C** mean kurtosis; **D** mean skewness. Lines connects median values over time, *p* values refer to interaction (time x group)

The mean amplitude and mean standard deviation had different trajectories in control and pooled HSL groups between T2 and T3 (*p* < 0.001 for interaction in both cases), but not between T1 and T2 (*p* = 0.86 and *p* = 0.95, respectively). Mean kurtosis and mean skewness had different trajectories between T1 and T2 and between T2 and T3 (*p* < 0.001 for all interactions) (Fig. 6, Additional file 1: Figs. S6 and S7). At T3, a higher proportion of control animals had suppressed background or suppression-burst patterns than did HSL-treated animals (*n* = 8/22; 100 vs. 63.6%, *p* = 0.046) (Additional file 1: Fig. S8). No animal had status epilepticus at T3.

### Gene expression

**Fig. 7:**
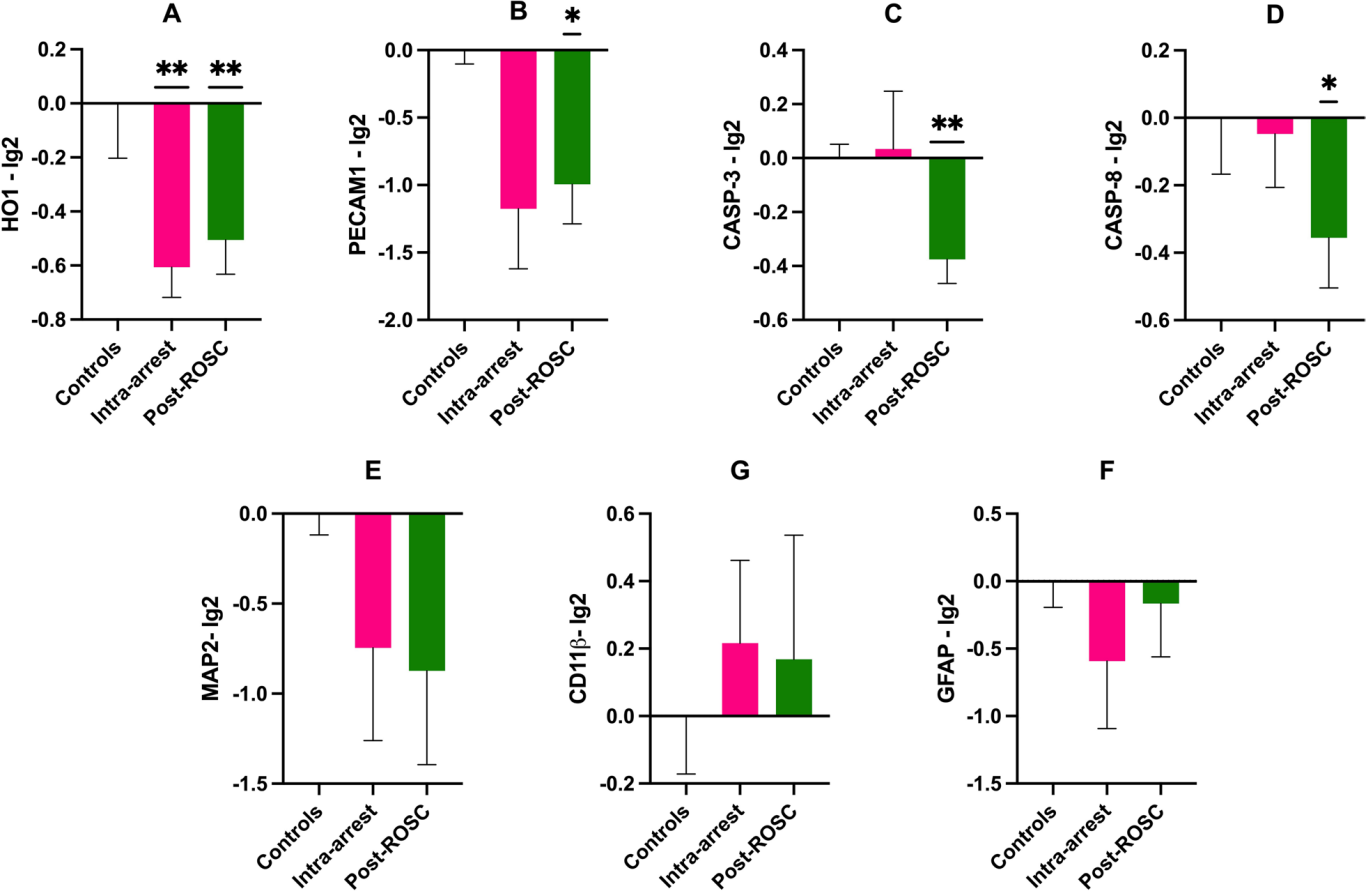
Gene expression in parietal cortex (Wilcoxon test). **A** *HO-1*; **B** *PECAM-1*; **C** *CASP-3*; **D** *CASP-8*; **E** *MAP2*; **F** *GFAP*; and **G** *CD11ß*. Mean values SEM. For controls *n* = 4, Intra-arrest *n* = 5; Post-ROSC *n* = 7. *p < 0.05; **p < 0.01 versus controls

The expression of *HO-1* was reduced in both HSL groups compared to controls (*p* < 0.01, Fig. 7A). *PECAM-1* expression was lower in the Post-ROSC group than in the controls (*p* = 0.01) but not in the Intra-arrest group (*p* = 0.05, Fig. 7B). Apoptosis-related *CASP-3* and *CASP-8* were lower in the Post-ROSC group than in the controls (*p* < 0.01 and *p* = 0.04, Fig. 7C, D). There were no statistically significant differences in the expression of *MAP2*, *CD11ß*, or *GFAP* between groups (Fig. 7E–G and Additional file 1: Table S2).

## Discussion

Our results demonstrate that infusion of HSL during CPR and/or early after ROSC has several effects: A—reduces norepinephrine requirements in the first 12 h after ROSC; B—mitigates brain and cardiac injury as indicated by a decrease in injury-related biomarkers and reduced cortical expression of brain injury-related genes; and C—was associated with different cerebral activity, as indicated by different EEG findings. Our experimental model was designed to achieve a maximum tolerated HSL infusion; despite reduction in the infusion rate in the second half of the observation period because of pre-set safety limits, HSL treatment remained feasible for the duration of the experiment. Our experiment was conceived based on previous report that similar no-flow time could provoke a significant brain injury while maximizing the ROSC rate [21].

Our findings are in line with those from a study by Stevin et al. [17], which showed that the infusion of molar HSL for 120 min in rabbits after ROSC increased the proportion of animals with pupillary reactivity at 2 h, reduced biomarkers of brain injury (astrocyte lysis marker S100β), improved hemodynamics (MAP, cardiac output, and left ventricular surface shortening fraction), and improved brain mitochondrial function. Similar to our results, HSL influenced lactatemia, natremia, and kalemia, and pH increased in HSL-treated animals [17]. However, the rabbits in the study by Stevin et al. [17] were treated with a higher HSL dose than that used in the present study (i.e., 80 µmol/Kg/min vs. 30 µmol/Kg/min) for a shorter period of time (i.e., 2 vs. 12 h), and the origin of CA (i.e., asphyxial vs. electrical) and CPR techniques differed. Furthermore, animals were not resuscitated to a minimal target MAP threshold and no targeted temperature management was used: By implementing these two factors, we limited the possible confounding effects of a different CPP and coronary perfusion pressure across groups when interpreting the organ damage. The demonstration of short-term safety and the reproducibility of certain benefits in two different preclinical models should pave the way to a clinical study assessing the physiological effects of HSL in this setting. In another trial, Miclescu et al. [22] compared solutions of methylene blue combined with HSL (0.63 M), normal saline, or hypertonic saline dextran in a pig model of CA. Although no group received HSL alone as an intervention, and the observation period lasted only 4 h, the methylene blue-HSL group had lower concentrations of CK-MB and troponin I compared to the methylene blue-normal saline group; in addition, the methylene blue-HSL solution had an alkalinizing effect. In contrast, there were no differences in plasma levels of protein S100β in the three groups at 4 h [22].

HSL-treated animals needed less vasopressor support and had a greater urine output despite similar fluid balance. The increase in the pH may have contributed to ameliorate the effects of endogenous and exogenous catecholamines [23]. On the other hand, despite a significant reduction in the myocardial injury as shown by the lower plasma level of troponin I at 6 h and 12 h in HSL-treated animals, there were no differences in cardiac output or cardiac power output. Previous studies suggested that lactate infusion may decrease myocardial injury after ischemia–reperfusion [24, 25]. One can reasonably hypothesize that the cardiac reserve of these previously healthy pigs was sufficient in conditions of deep anesthesia and hypothermia to provide hemodynamic tolerance to the initial insult. It remains possible that, after discontinuation of TTM and sedation, with an increase in systemic and myocardial VO_2_, myocardial dysfunction could become clinically significant. Importantly, no significant differences were found between the Intra-arrest and the Post-ROSC groups, suggesting that a clinical trial could be designed to test this therapy only in patients with a sustained and stable circulation.

After CA, there was a rapid increase in plasma GFAP levels, consistent with observations in patients [26]. Of note, there was a significant reduction in plasma GFAP in HSL groups at 6 and 12 h post CA. While traditionally considered a marker of astrogliosis, blood GFAP levels have been suggested to reflect disruption of the blood–brain barrier in acute CNS injury [27] and in highly inflammatory conditions, such as coronavirus disease 2019 (COVID-19) [28]. As a matter of fact, GFAP is a key constituent of the astrocytic endfeet enveloping the blood–brain barrier which, when damaged, may release GFAP directly into the bloodstream. A reduction in blood–brain barrier damage following treatment may thus underpin the neuroprotective effects of HSL. Although only NSE is recommended as an outcome predictor after CA in clinical practice [29], other biomarkers have been suggested and may be more accurate [30]. This study is among the first large animal studies of CA in which NFL has been measured during a 12 h observation period [31]. In contrast to our findings for GFAP, there was no statistically significant effect of HSL on NFL and NSE levels, although there was a trend toward lower concentrations of these biomarkers in the HSL groups. Whereas GFAP rapidly increases in the blood of patients with CA [32], NFL and NSE levels peak between 24 and 72 h after ROSC [32, 33]. As such, it is possible that with a more prolonged observation period (i.e., 24–48 h), there may have been different trajectories of these biomarkers (i.e., NSE and NFL) among the study groups.

Although there were no significant differences in ICP, PbtO_2_, and regional CBF when HSL was infused, ICP and PbtO_2_ remained within normal ranges, suggesting that HSL had a limited impact on these variables. Nonetheless, heterogeneous ICP values have been reported in CA survivors and we cannot make any inference about the possible utility of hypertonic infusions in these subjects [34–36]. Moreover, the increase in pH and decreased cerebral temperature in HSL-treated animals might, theoretically, have increased the Hb affinity for oxygen by shifting the Hb dissociation curve to the left, reducing interstitial oxygen availability and, therefore, absolute PbtO_2_ values. Despite the metabolic effects of HSL, there were no differences in cardiac output or CPP, suggesting that the overall cerebral DO_2_ did not differ during the observation period. Although the cerebral metabolites explored using CMD were similar in the three groups, except for an increase in cerebral lactate, the arterio-jugular difference in lactate and glucose suggests that an increased uptake of lactate with a glucose sparing effect was present at some time points in the HSL groups, thus highlighting the possible metabolic shift between different substrates in case of acute injury. One could also argue that the use of deep sedation and TTM decreased the cerebral metabolic rate [37, 38], potentially resulting in an underestimation of the impact of HSL on cerebral function. Although the increased EEG activity does not equate to a better prognosis, intracerebral EEG recordings suggested an earlier recovery of the background rhythm, which may be secondary to a more rapid restoration of cerebral metabolism or perfusion. HSL has been shown to improve mitochondrial function [17], and we noted reduced expression of genes related to oxidative stress (*HO-1*), endothelial function, and in the Post-ROSC group apoptosis (CASP-3 and CASP-8). There were no significant differences for neuronal, microglial, and astrocyte related genes. Plasma GFAP, NFL, and NSE concentrations are indicative of damage to existing cerebral cells at the time of injury, but gene expression analyses reflect cell reaction *after* injury. The lack of effects of HSL on these parameters suggests that later time points should be investigated to observe possible reparative/compensative effects induced by HSL. Of note, one animal in a HSL-treated group experienced a second arrhythmic event during the observation period. Although this event was triggered by the manipulation of the pulmonary artery catheter during hypothermia (7 h after ROSC), not routine practice in the clinical setting, we cannot exclude that the lower potassium levels induced by the treatment (3.52 mEq/l) might have increased the chances of an arrhythmic event. Nevertheless, together, our data suggest that the favorable effects on brain damage of HSL infusion in CA are related to multiple mechanisms rather than a single one.

Our study has several strengths: First, our model of CA provided a good survival rate but also severe post-resuscitation vasoplegia and cerebral damage. Second, many different physiological parameters, including multimodal neuromonitoring variables, intracranial EEG, and cardiac output, were continuously recorded. Third, our observations were conducted over a relatively long period after ROSC, thus capturing physiological modifications produced by both primary (i.e., minutes) and secondary (i.e., hours) injuries after hypoxic ischemic brain injury.

Our study also has several limitations: First, we selected an arbitrary dosage of the intra-arrest bolus of HSL, and this might have been insufficient to elicit a biological effect. Nevertheless, it is possible that a different experimental design with longer CA duration would increase the possibility for a clinical impact of the intra-arrest bolus of HSL. Second, swine have different lactate metabolism compared to humans and this might limit the applicability of the present results to other species. Nevertheless, the cardiovascular system and brain architecture of swine closely resemble those of humans, providing a reliable experimental model. Third, no ultrastructural, morphological, or histological analyses were performed to confirm the decreased organ injury in the HSL-treated groups. Fourth, although different areas of the brain might exhibit different susceptibilities to hypoxic ischemic injury, gene expression was explored only in the parietal cortex. Fifth, regional multimodal neuromonitoring was used, and caution should therefore be taken in generalizing our results to the brain parenchyma as a whole. However, this approach is derived from current neurocritical care practice in humans; additionally, as the hypoxic event is rather homogenous, regional monitoring should still be able to detect relevant changes in key physiological variables. Sixth, no additional hemodynamic monitoring (i.e., pressure/volume curves or echocardiography) was performed. However, we wanted to minimize the possibility of inducing a ventricular arrhythmia in the post-resuscitation phase, and the use of ultrasound was limited by the ventral position of the animal after ROSC.

## Conclusions

In this experimental, large animal model of CA, administration of HSL, started during CPR or after ROSC, was associated with reduced norepinephrine requirements and lower circulating levels of biomarkers of cardiac and brain injury.

## Supplementary Information

**Additional file 1**. Supplementary data.

## Data Availability

The datasets used during the current study are available from the corresponding author on reasonable request.
