# Supplementary material for: Hypertonic sodium lactate infusion reduces vasopressor requirements and biomarkers of brain and cardiac injury after experimental cardiac arrest
Source: Crit Care. 2023 Apr 22;27:161. doi: 10.1186/s13054-023-04454-1 (PMC10122448; doi:10.1186/s13054-023-04454-1)
Supplement: Supplementary file 1 — Additional file 1. Supplementary data. [file 13054_2023_4454_MOESM1_ESM.docx]

**Hypertonic sodium lactate infusion reduces vasopressor requirements and biomarkers of brain and cardiac injury after experimental cardiac arrest**

**Online supplementary data**

**Additional Protocol Information**

*Experiment location:*

All the experiments were conducted in the Experimental Laboratory of Intensive Care of the Free University of Brussels (ULB), located within the university's animal laboratory at the Erasme campus in Anderlecht, Belgium (Agreement number: LA1230336; Head of the Laboratory: Professor Jacques Creteur).

*Participating staff:*

This series of experiments was conceived to test different medical therapies during and after CA. They were conducted by two certified (FELASA-A, FS and FELASA-B, FA) intensivists under the supervision of the facility veterinarian.

*Animal origin:*

Provider: Ra-SE Genetics Holding N.V. (Belgium, agreement number: BE40012565)

*Housing and husbandry:*

An automatic water delivery system is available in each cage, as well as playballs. The day-night cycle is respected and hygiene, nutrition, available space, and temperature in the cage are standardized in accordance with Annex 4 of the Belgian Royal Decree of May 29, 2013. The animals stayed in a cage for the minimum possible time, in all cases less than 7 days.

*Monitoring of distress, pain, and humane endpoints:*

All the experiments were conducted under deep general anesthesia and awakening of the included animals was not planned. During the experiment, the absence of pain or distress as well as the depth of the anesthesia was achieved with continuous monitoring of cardiac frequency and blood pressure variations during potentially painful procedures or, during the observation phase, via a dedicated transitory pain stimulus test (once every hour), with the appreciation of increased jaw tone and of corneal reflexes.

**Detailed protocol description**:

On the day of the experiment, the animal (swine, *Sus Scrofa Domesticus*) having fasted for 12 hours with free access to water, was initially sedated in the cage with an intramuscular injection of midazolam (1 mg/kg, Mylan, White Sulphur springs, WV, US) and ketamine (10 mg/kg, Dechra Be, Belgium) in the neck, and then placed supine on the operating table. Continuous electrocardiogram (ECG) monitoring was placed and after cannulation of a marginal ear vein with a 20-gauge catheter (Becton-Dickinson, Franklin Lakes, NJ, US), a continuous infusion of sufentanyl citrate (0.2-1 µg/kg/h, Jansen, Belgium) was started. A femoral 4.5 Fr arterial catheter (Vygon, France) was inserted in the right femoral artery using ultrasound guidance (Vivid E9, GE Healthcare, Chicago, IL, US) and connected to a pressure transducer (True Wave, Edwards, Irvine, CA, US). After sequential intravenous injections of 1 mg atropine sulfate (Sterop, Belgium), 3 µg/kg of sufentanyl citrate, and 1.2 mg/kg of rocuronium (Esmeron, MSD, Kenilworth, NJ, US), an 8-mm inside diameter endotracheal tube (Medtronic, Dublin, Ireland) was placed, and mechanical ventilation was started in controlled volume mode (Primus, Drägerwerk AG & Co., Germany) with a tidal volume of 8 ml/kg, 5 cmH_2_O of positive end-expiratory pressure (PEEP), fraction of inspired oxygen (FiO_2_) of 1, and inspiratory to expiratory ratio of 2, with a square wave flow pattern. A 1% mixture of inspired sevofluorane (Sevoflo, Abbott, Chicago, IL, US) was started. Ventilatory parameters were adjusted to ensure a PaCO_2_ between 35 and 45 mmHg and a PaO_2_ >70 mmHg, with the minimally required FiO_2_. A continuous infusion of rocuronium (1-4 mg/kg/h) and sufentanyl citrate (3.5 µg/kg/h) was started and 2g of amoxicillin-clavulanate (Sandoz, Basel, Switzerland) administered as a slow intravenous bolus.

A 14-Fr Foley catheter was placed surgically thorough a midline incision in the lower abdomen to measure urine output and the parietal layers were sutured separately. With the help of ultrasound guidance, a triple lumen central venous catheter (Teleflex, Wayne, PA, US) was placed in the right external jugular vein and a 6-Fr introducer (TERUMO Europe, Leuven, Belgium) placed proximally in the same vein to enable pacing wire insertion. A single lumen central venous catheter (Teleflex, Wayne, PA, US) was inserted upstream in the left internal jugular vein and advanced until shortly before resistance to allow sampling of brain effluent blood. Lastly, an 8.5-Fr introducer was placed in the external left jugular vein and a 7-Fr continuous cardiac output pulmonary artery catheter (Edwards, Irvine, CA, US) placed in the pulmonary artery and advanced to acquire pulmonary arterial pressures, continuous cardiac output, and mixed venous oxygen saturation (SvO_2_) displayed via a dedicated monitor (Vigilance II monitor, Edwards, CA, US). The fluid infusion rate was adjusted to maintain an arterial pulse pressure variation (PPV) <14%. The animal was then placed prone, dorsal ECG electrodes positioned, and the bed angled to achieve a 30° anti-Trendelenburg position.

*Neurosurgical procedure*

The forehead of the animal was shaved and disinfected with povidone iodine (iso-betadine, Meda Pharma, Sweden). At approximately 0.5 cm from the midline, a reverse mirrored F incision was executed on both sides with the aid of an electric scalpel, and two cutaneous flaps were fixed to expose the frontal bones using suture wire. Using a battery alimented drill (NM100, Ruijin Medical Instruments, China), six burr holes were drilled in the skull, two in the center of the area covered by the cutaneous flap and four on the main incision lines. In the rostral burr holes, a 10-mm length microdialysis catheter (CMA 20, CMA microdialysis, Sweden) was inserted and engulfed in bone wax, and a continuous perfusion with central nervous system-like perfusion fluid (CNS fluid, CMA microdialysis, Sweden) at the rate of 0.3 µl/min was started to allow sampling every 60 minutes. In the other burr holes, four bolts (Bolt-drill kit CH5, Raumedic AG, Germany) were placed for insertion of a custom-built needle to open the dura mater with minimal trauma. On the right side, a multifunctional probe recording cerebral temperature, intracranial pressure (ICP), and brain oxygen pressure (PbtO_2_; Neurovent-PTO2, Raumedic AG, Germany) was inserted and connected to a dedicated monitor (MPR2 logO datalogger, Raumedic AG, Germany). On the left side, a laser Doppler flowmetry probe was placed (Oxyflow 4000, Oxford Optronic, UK) to estimate cerebral regional blood flow (CBF). In each of the caudal bolts, a 5-contact intracranial stereoelectroencephalography (sEEG) wire (Microdeep 5 SEEG electrode, Dixi medical, France) was inserted, connected to an acquisition device (Refa 40, TMSi, Netherlands) with transfer to an acquisition software (NOTOCORD-Hem 4.4.0.2, Notocord systems, France). The animal was then turned supine, and the head positioned beyond the edge of the table, kept in place by a perforated, removable table headrest.

*Cardiac arrest procedure*

After placing the automatic chest compression system (LucasIII, Jolife AB/Stryker Lund, Sweden) and ECG electrodes, two adhesive defibrillating pads were placed in latero-lateral position (Stad-padzII, Zoll Medical Corporation, Chelmsford, MA, US) and connected to a biphasic defibrillator (Zoll M series, Zoll Medical Corporation, Chelmsford MA, US). A pacing catheter was advanced through the left jugular vein introducer to induce ventricular fibrillation (VF), which was confirmed by the appearance of the typical ECG waveform and the abrupt decrease in arterial blood pressure. Mechanical ventilation was then stopped, and the animal left untreated for 10 minutes (no-flow time). After this period, chest compressions were started at the rate of 100/minute for 5 minutes and ventilation was resumed with a FiO_2_ of 1. After one minute, an intravenous injection of epinephrine (30 µg/kg, Sterop, Belgium) was administered through a dedicated line, which was then flushed with 10 mL of crystalloid, and anesthetic gas flow resumed. At the end of the 5-min period, after confirmation of the persistence of VF, a 4 J/kg biphasic electric countershock was delivered. CPR was restarted for an additional minute in case of unsuccessful return of cardiac activity and another shock was delivered every minute along with a second dose of epinephrine for a total CPR time of 7 minutes. Return of spontaneous circulation (ROSC) was considered to have been achieved if the rhythm remained compatible with the presence of a mean arterial pressure (MAP) >65 mmHg for at least 20 minutes. If the animal failed to achieve ROSC after 10 shocks, it was considered as deceased. If ROSC was achieved, the animal was placed prone and observed for 12 more hours.

All animals were treated with targeted temperature management (TTM) using an external feedback device aimed at maintaining a temperature of 34 °C (Arctic Sun 5000, Bard Medical Division, Murrey Hill, NJ, US). At the end of the observation period, or if a major protocol complication occurred, the animals were sacrificed under deep anesthesia with the injection of 80 mEq of potassium chloride via the central venous line.

**Table S1:** the ARRIVE checklist.


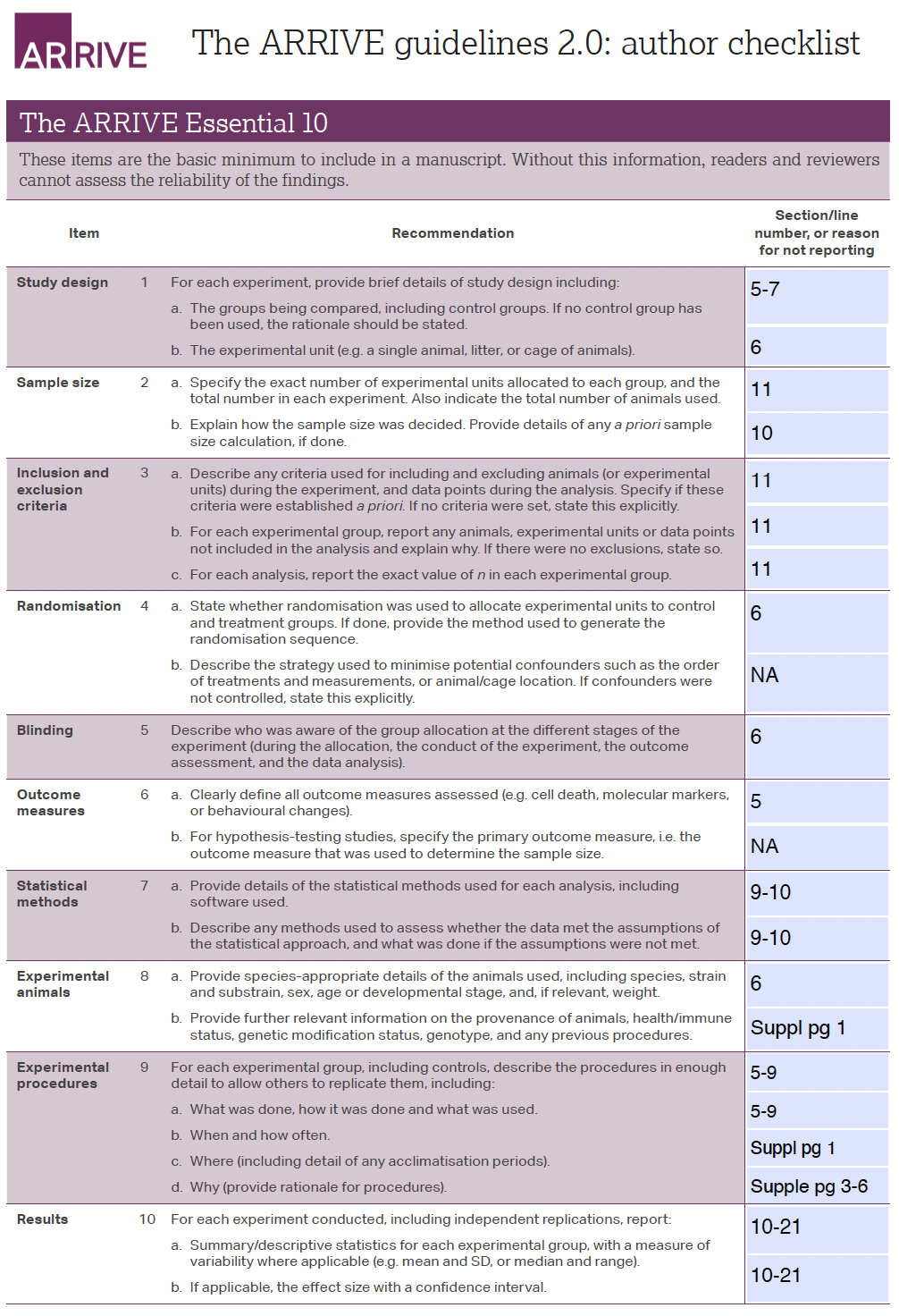


**Table S2**: Primers used for real-time reverse transcription polymerase chain reaction. National Center for Biotechnology Information (NCBI).

| **Gene** | **NCBI** | **Forward primer** | **Reverse primer** |
| --- | --- | --- | --- |
| *RPL27* | [NM_001097479.1](https://www.ncbi.nlm.nih.gov/nuccore/NM_001097479.1) | CATGCCCACAAGGTACTCCG | CTTGACCTTGGCCTCTCGTC |
| *GFAP* | [NM_001244397.1](https://www.ncbi.nlm.nih.gov/nucleotide/346986348?report=gbwithparts) | TGCAGATCCGAGAAACCAGC | GCAGGCTTAATGCTGATGGC |
| *CD31* | [NM_213907.1](https://www.ncbi.nlm.nih.gov/nucleotide/47522669?report=gbwithparts) | CTCAAGAAATCCAAGGCCAAGC | TCAGCTTTCCGGATTTCACTGT |
| *Nfe2l2* | [XM_003133500.6](https://www.ncbi.nlm.nih.gov/nucleotide/1191841847?report=gbwithparts) | TTCGAAGTCAGAGTCGGCTG | GAATGTGGGCTACCTGGGAA |
| *MAP2* | [XM_021075018.1](https://www.ncbi.nlm.nih.gov/nucleotide/1191841577?report=gbwithparts) | TTGTGACTGCTGGCTGGAAT | CTTGCAGACACCTCCTCTGG |
| *Caspase 3* | [NM_214131.1](https://www.ncbi.nlm.nih.gov/nucleotide/47523065?report=gbwithparts) | TTCTTCAGAGGGGACTGCTG | CCTCGGCAGGCCTGAATTA |
| *Caspase 8* | [NM_001031779.2](https://www.ncbi.nlm.nih.gov/nucleotide/284519711?report=gbwithparts) | CCAGGATTTGCCTCCGGTTA | CAGGCTCAGGAACTTGAGGG |
| *Hmox-1* | [NM_001004027.1](https://www.ncbi.nlm.nih.gov/nucleotide/51592104?report=gbwithparts) | TACCGCTCCCGAATGAACAC | GTCACGGGAGTGGAGTCTTG |
| *CD11b* | [U40072.1](http://www.ncbi.nlm.nih.gov/nuccore/U40072.1) | AGAAGGAGACACCCAGAGCA | GTAGGACAATGGGCGTCACT |

**Table S3:** CPR-related variables.

|  | **Group** | | | ***p* value*** |
| --- | --- | --- | --- | --- |
| **Variable** | **Control** | **Intra-arrest** | **Post-ROSC** |  |
| Total CPR time, seconds | 300 (300-360) | 300 (300-360) | 300 (300-360) | 0.82 |
| Countershock, n | 1 (1-2) | 1 (1-2) | 1 (1-2) | 0.78 |
| Epinephrine dose, mg | 1.4 (1.4-1.4) | 1.4 (1.4-1.4) | 1.4 (1.4-1.4) | >0.99 |
| Max etCO_2_ first minute, mmHg | 32 (27-35) | 28.5 (27-33.8) | 27 (24-37) | 0.65 |
| Arrhythmia within 30 minutes after ROSC, % | 9.1 (3.1-23.5) | 12.2 (4.8-27.3) | 15.2 (6.6-30.9) | 0.93 |

*ANOVA. CPR=cardiopulmonary resuscitation; etCO_2_=end-tidal CO2; ROSC=return of spontaneous circulation.

**Figure S1:** Proportions of animals in the three groups with a positive arterio-jugular difference for glucose (A) and lactate (B)

**
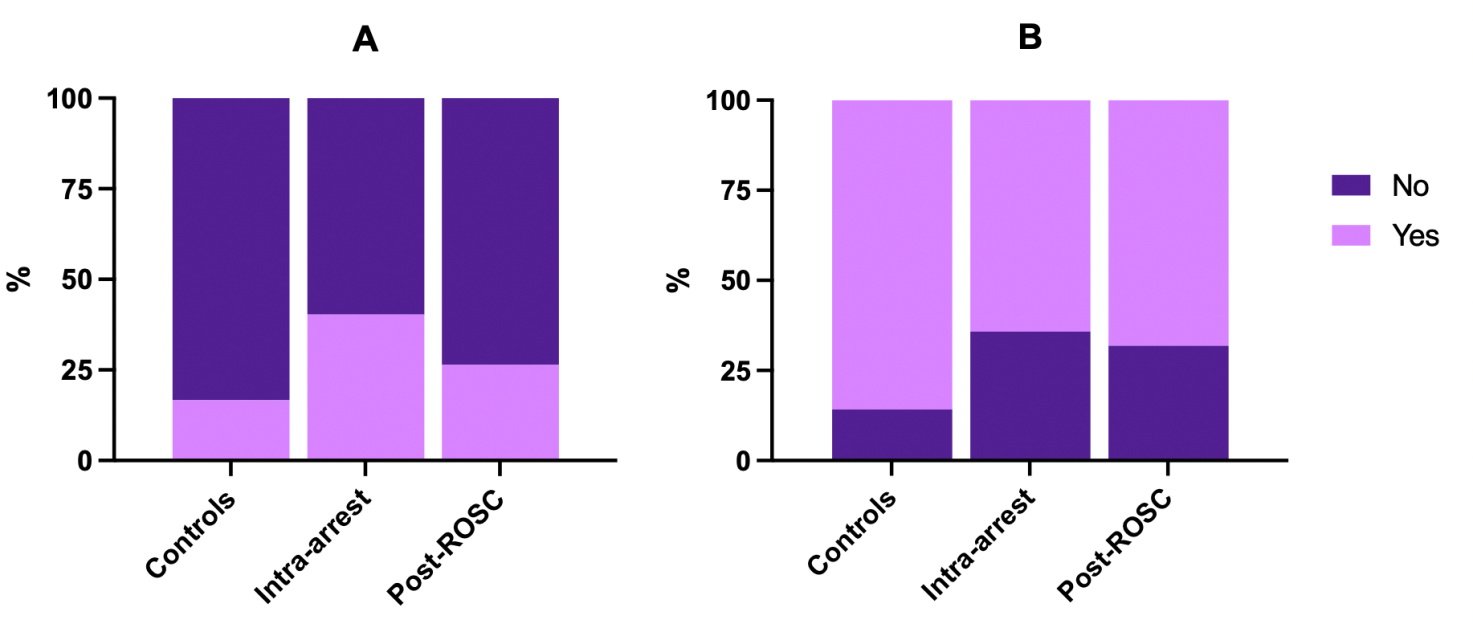
**

**Table S4:** Main hemodynamic variables.

| **Variable** | ***p* value*** |
| --- | --- |
| MAP, mmHg | 0.86 |
| HR, bpm | 0.90 |
| CVP, mmHg | 0.33 |
| SvO_2_, % | 0.22 |
| CO, l/min | 0.94 |
| PPV, % | 0.33 |
| PAOP, mmHg | 0.08 |
| CPO, Watt | 0.87 |
| DO_2_, ml/min | 0.96 |
| VO_2_, ml/min | 0.80 |
| OER, % | 0.53 |

*General mixed model: p values refer to interaction between time and groups.

MAP=mean arterial pressure; HR=heart rate; CVP=central venous pressure; SvO2=mixed venous oxygen saturation; CO=cardiac output; PPV=pulse pressure variation; PAOP=pulmonary artery occlusion pressure; CPO=cardiac power output; DO_2_=oxygen delivery; VO_2_=oxygen demand; OER=oxygen extraction ratio.

**Figure S2**: Diuresis (A) and fluid balance at T3 (B). Data shown as means and standard error of the mean (SEM). *= significant difference between Controls and Intra-arrest group, *°= significant difference between Controls and both HSL treated groups.


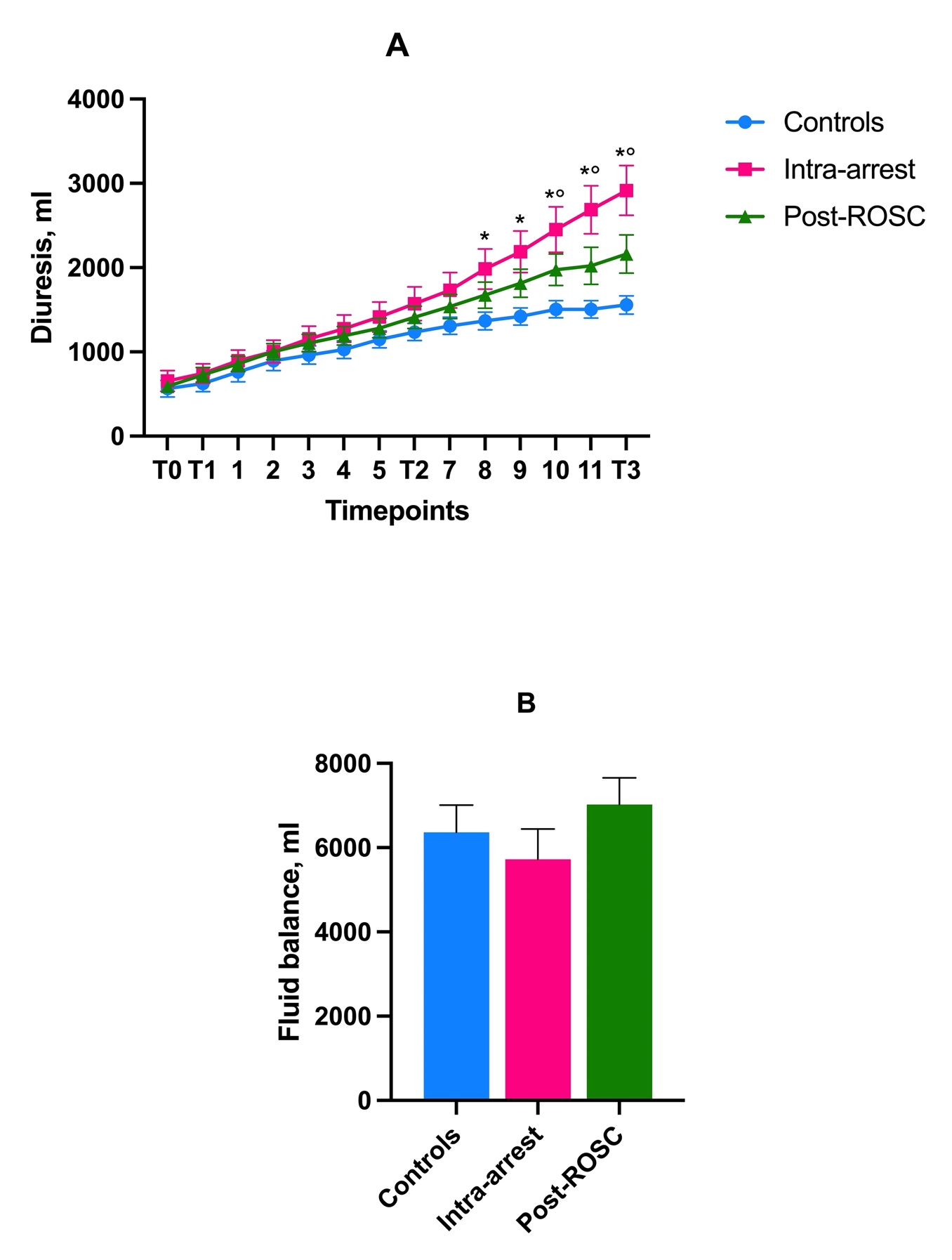


**Figure S3: T**ime-course of multimodal neuromonitoring variables. A=intracranial pressure (ICP); B=brain tissue oxygen pressure (PbtO_2_); C=cerebral perfusion pressure (CPP); D=cerebral blood flow (CBF). Data shown as medians with 95% confidence interval (CI).


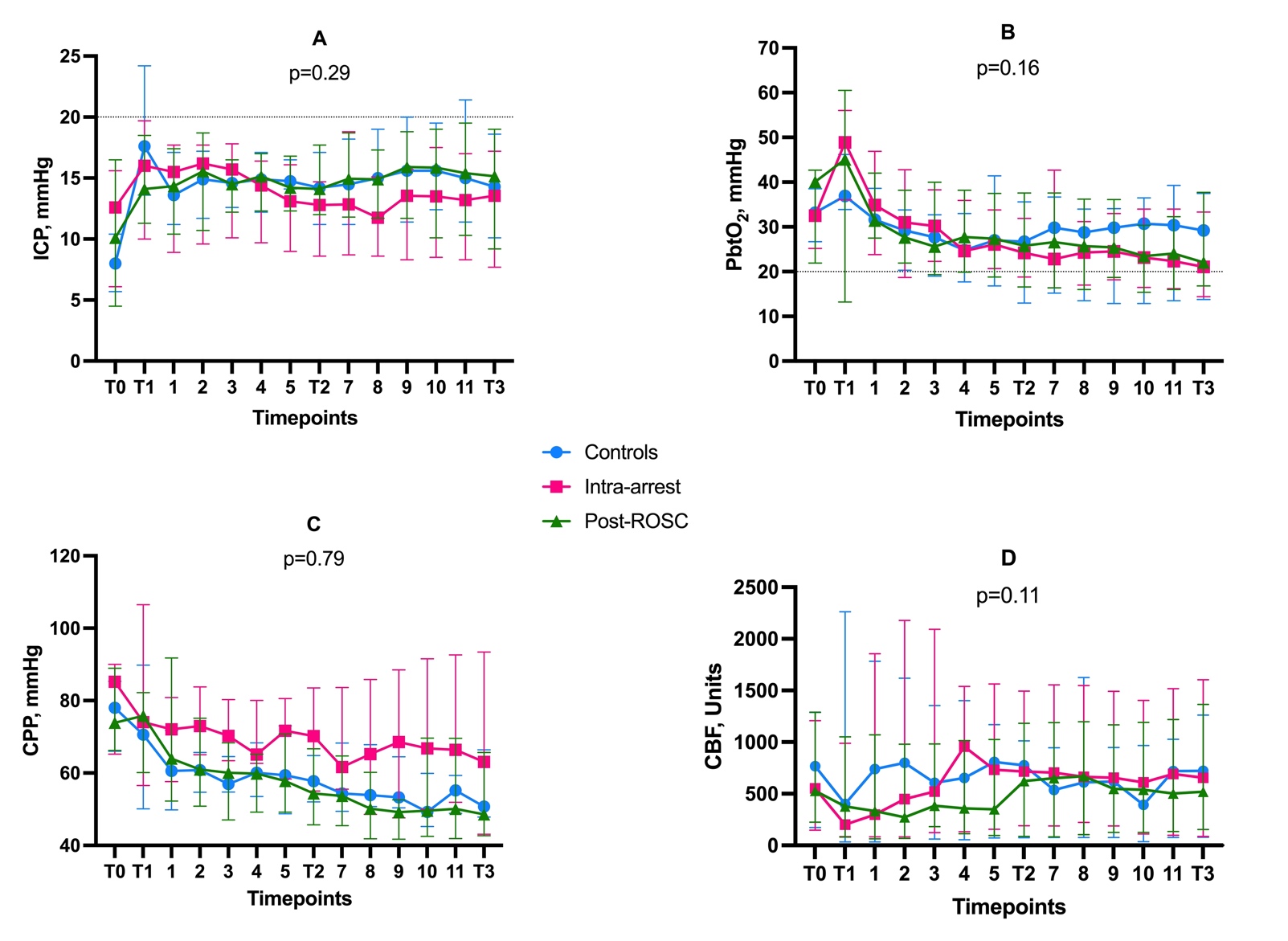


**Figure S4:** Time-course of cerebral microdialysis variables. A=lactate; B=glucose; C=pyruvate; D=lactate to pyruvate ratio; E=glutamate; F=glycerol. Data shown as medians with 95% CI (A-D, F) or means with SEM (E).

**
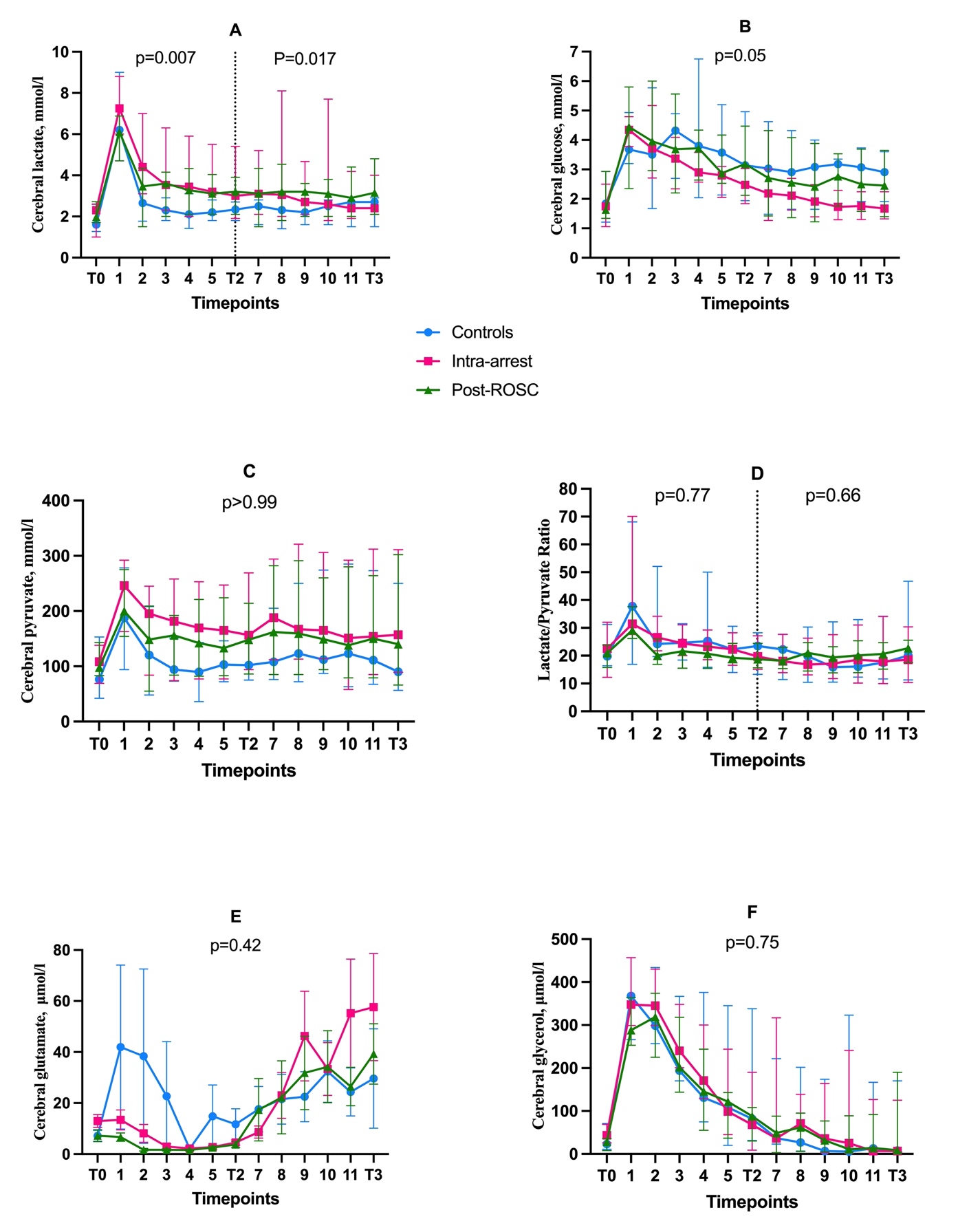
**

**Figure S5:** Time-course of measured biomarker concentrations: A=aspartate aminotransferase (AST); B=alanine aminotransferase (ALT); C=creatinine; D=urea; E=lactic dehydrogenase (LDH); F=creatine phosphokinase (CPK); G=alkaline phosphatase (ALP).


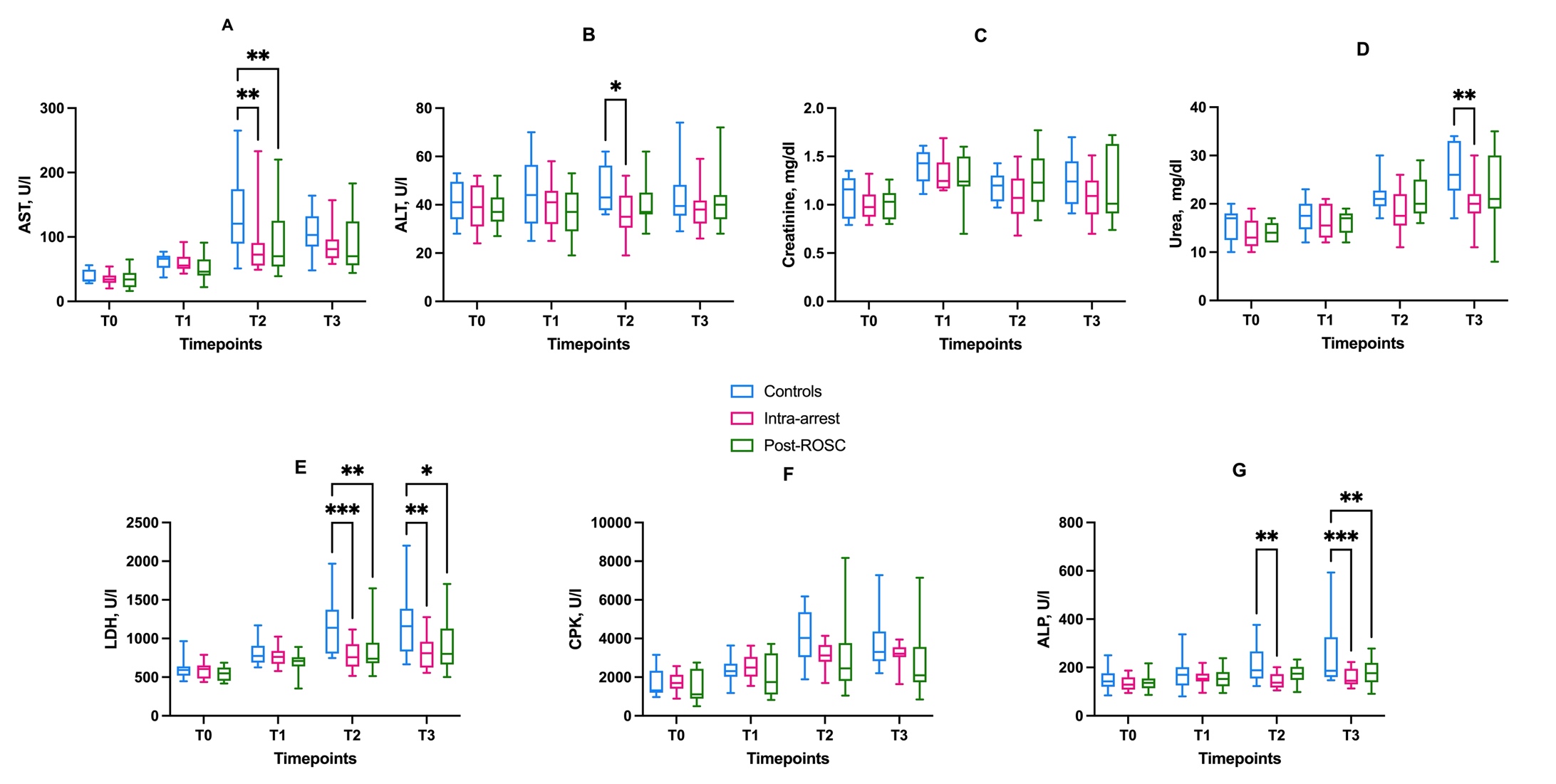


**Figure S6:** Evolution of EEG mean amplitude over time in controls and grouped hypertonic sodium lactate (HSL) recipients. Each row represents median values after remove of outliers (ROUT with Q=1%) at each timepoint.


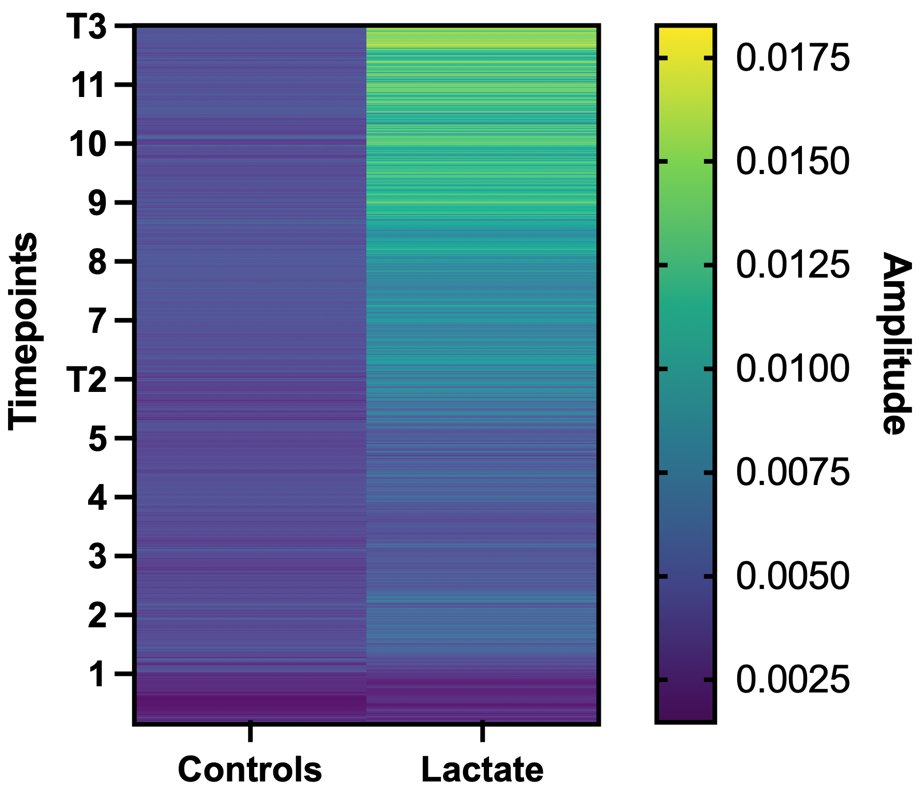


**Figure S7:** EEG evolution: A=mean amplitude; B=mean standard deviation; C=mean kurtosis; D=mean skewness. Lines connect median values over time.


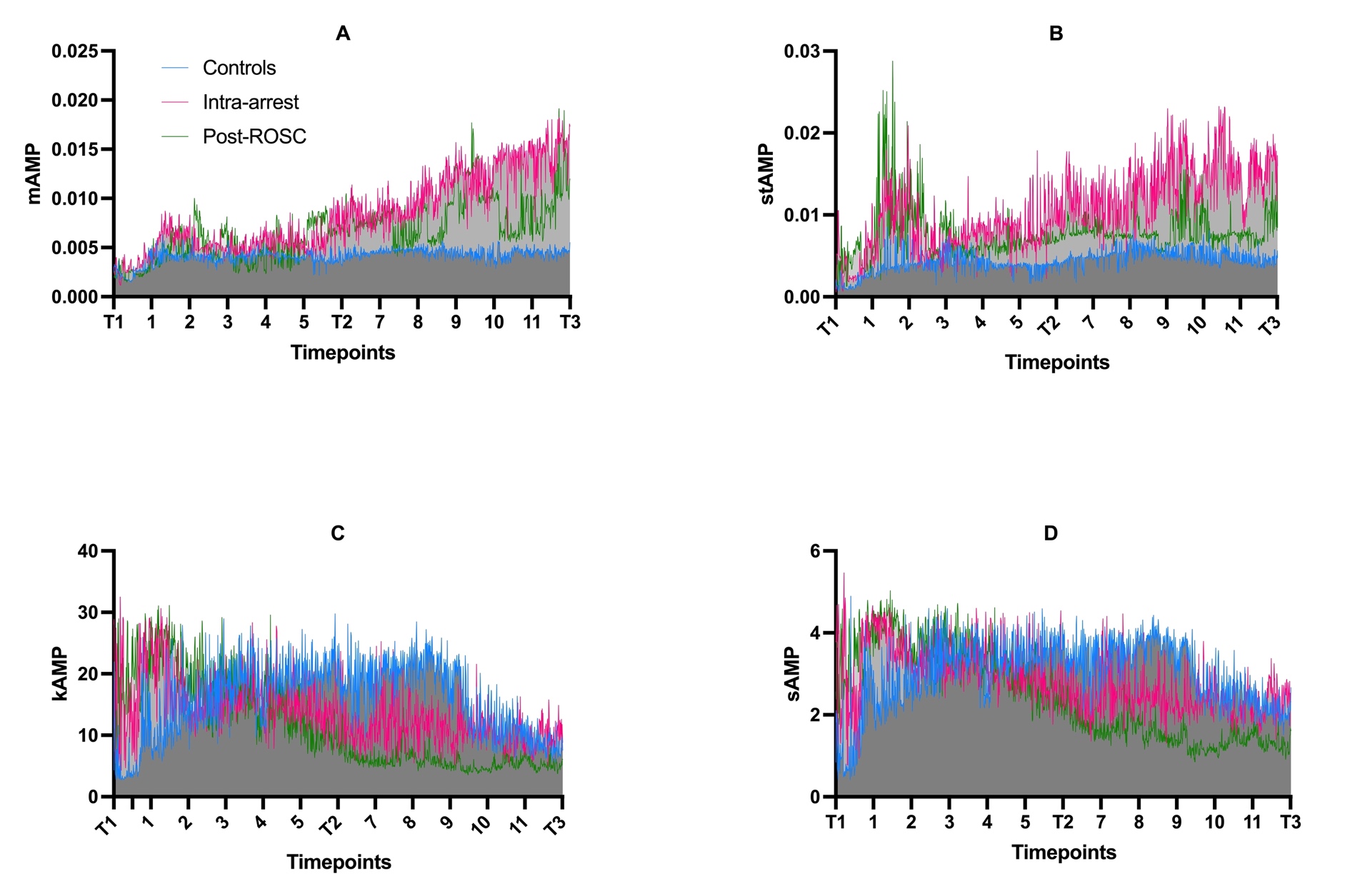


**Figure S8:** Proportion of animals with a suppressed background (S) or suppression-burst (SB) EEG pattern at T3 in the three groups.

**
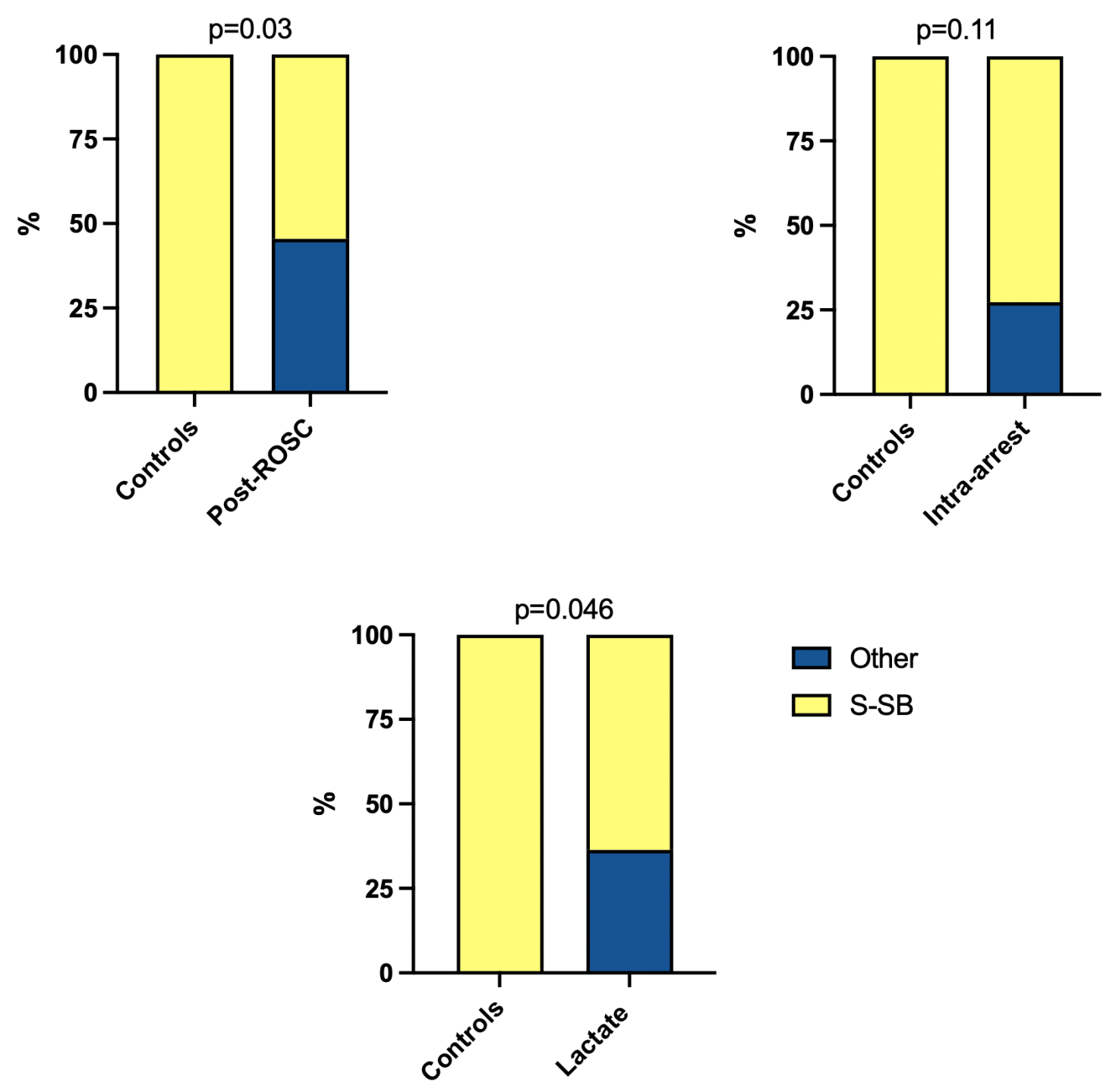
**

**Table S2:** Gene expression in the parietal cortex. *p* values refer to the Wilcoxon test of Groups compared to controls.

|  | *p* value | |
| --- | --- | --- |
|  | **Intra-arrest** | **Post-ROSC** |
| ***MAP2*** | 0.31 | 0.21 |
| ***GFAP*** | 0.30 | 0.68 |
| ***CD11b*** | 0.42 | 0.66 |
| ***PECAM1*** | 0.05 | 0.01 |
| ***CASP3*** | 0.88 | 0.005 |
| ***CASP8*** | 0.77 | 0.04 |
| ***HO-1*** | 0.005 | 0.007 |

MAP2= microtubule-associated protein 2; GFAP=glial fibrillary acid protein; *CD11ß* =cluster of differentiation molecule 11ß; *PECAM1=* platelet and endothelial cell adhesion molecule 1; *CASP3* and *CASP8=* caspase 3 and 8; HO-1= heme oxygenase 1.
